# Psychiatric symptoms in a female with subacute combined degeneration of the spinal cord (SCD): a case report

**DOI:** 10.1186/s12888-023-04631-0

**Published:** 2023-03-01

**Authors:** Yinlin Zhang, Huirong Luo, Xueqian Wang, Haitang Qiu, Hao Ren, Anhai Zheng, Qinghua Luo

**Affiliations:** 1 Department of Psychiatry, the First Affiliated Hospital of Chongqing Medical University, No. 1 Youyi Road, Yuzhong District, Chongqing, 400016 China; 2 Department of Psychiatry, Changshou Third People’s Hospital, Changshou, 401231 Chongqing, China

**Keywords:** Subacute combined degeneration of the spinal cord (SCD), Vitamin B12, Psychiatric symptoms, Megaloblastic anemia, Case report

## Abstract

**Background:**

Subacute combined degeneration of the spinal cord (SCD) is mainly caused by deficiency of Vitamin B12 and characterized by deep hypoesthesia, sensory ataxia and spasmodic paralysis of lower limbs. SCD often accompanies with megaloblastic anemia. Psychiatric symptoms could be the initial manifestations of SCD by lack of Vitamin B12, but are rarely considered secondary to physical discomfort and psychological factors in SCD. Additionally, treatment experience for psychiatric symptoms in SCD remains little reported.

**Case report:**

We presented a case of a 37-year-old female who complained of being persecuted and controlled for one week and thus was admitted to the psychiatry department. Before that, she had went through persistent paresthesia and numbness of her lower extremities for two-month. Low Vitamin B12 level and hemoglobin concentration, neurologic symptoms and bone marrow smear results supported the clinical diagnosis of SCD and megaloblastic anemia. With supplementation of Vitamin B12 and blood transfusion and short-term prescription of antipsychotics and antidepressants, physical symptoms were improved and psychological symptoms disappeared within 2 weeks.

**Conclusions:**

Psychiatric symptoms of SCD could be generated from lack of Vitamin B12, anemia and neurologic symptoms, where short-term use of antipsychotics and antidepressants may be effective.

## Background

Subacute combined degeneration of the spinal cord (SCD) is the most common neurological disorder caused by Vitamin B12 deficiency, which often accompanied with megaloblastic or pernicious anemia [1]. Psychiatric symptoms, such as hallucinations, irritability, depression and delirium and apathy, were noticed in SCD and presumed to be associated with Vitamin B12 deficiency [2]. However, this supposition is not enough to fully account for psychiatric symptoms. Instead, physical discomfort and anemia should also be considered. Here, we presented a case of SCD with psychiatric symptoms closely relevant to physical discomforts such as gait ataxia and sensory abnormalities. We tried to illustrate possible mechanisms of psychiatric symptoms occurrence: psychological, organic, or both in combination. We shared our experience of antipsychotics and antidepressants application to prompt further discussion.

## Case presentation

A 37-year-old female patient was admitted to psychiatric department with complaints of numbness and prickling sensations in the lower extremities for the past 2 months and being persecuted and controlled for one week. Prior to disease onset, she was a curtain retailer and could take care of herself. The numbness progressed gradually, from level of ankles to the waist over time. She experienced a depressed mood and loss of interest after the occurrence of these discomforts. She was taken care of by her husband thereafter. One month before she developed symptoms of low appetite, worry, anxiety and gait described as ‘stepping on cotton’. One week before she couldn’t walk, couldn’t help fearing and trembling and found her husband suspicious of controlling her. At our psychiatric evaluation, the patient reported that at first she only had numbness and prickling sensations on the lower extremities. Gradually she began to feel depressed, worried, and unable to raise interest in doing things, and increasingly worried. With persistence of symptoms, she developed fears and suspiciousness. Meanwhile, she presented with psychotic symptoms. She believed that her husband was hostile to her by clicking on the phone to manipulate her legs, making her feel pain and out of control. Additionally, after admission, she distrusted doctors and nurses. She believed that the doctors use their pens as manipulation buttons, the pressing of which could control her body and make her feel numb and uncomfortable. She was extremely suspicious and unable to calm down at night. The psychiatric examination showed decreased psychomotor activity, excessive worrying, despair and anxiety, difficulty in falling asleep, delusions of being controlled and mood-congruent persecutory delusions. Her insight was reduced. But the patient did not have deficits in memory, concentration, or orientation.

Before admission, she visited gastroenterology outpatient and received only Mosapride 5 mg orally three times per day for her low appetite and fatigue, which brought no improvement. Several days later, she went to the neurology outpatient for limb numbness. Examinations were arranged 10 days before admission. Blood routine showed red blood cell (RBC) of 1.31 × 1012/L and hemoglobulin (HGB) of 61 g/L while head CT, electroencephalogram (EEG), motor nerve conduction velocity and sensory nerve conduction velocity results were normal. However, she did not come back to doctor after the medical results being reported until her psychiatric symptoms emerged. Then she came to psychiatry outpatient and was immediately hospitalized. After admission, it was found that she kept a vegetarian diet for approximately 8 years. She denied any substance abuse or family history of psychiatric disorders. Neurological examination revealed impaired acupuncture and vibratory sensation and decreased muscle strength in lower extremities. Speculated diagnoses included SCD and delusional disorder, and systematic examinations were suggested.

**Fig. 1:**
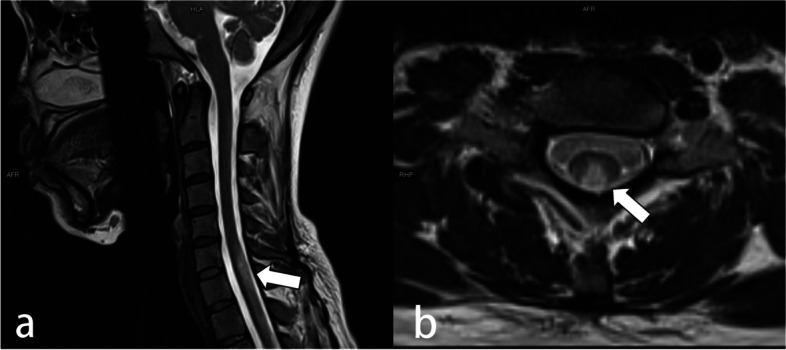
**A** Sagittal T2-weighted image showing abnormal hyperintense signal in the posterior aspect of the spinal cord (arrow) extending from the level of C6. **B** Axial T2-weighted image of the cervical spinal cord showing symmetric bilateral hyperintensity in the dorsal and lateral columns(inverted V sign) (indicated by white arrow)

Laboratory tests showed Vitamin B12 < 36.9 pmol/L [reference range 145.0–569.0 pmol/L]), RBC 1.91 × 10^12^/L, Mean Corpuscular Volume (MCV) 113.6 fl [reference range 82-100 fl]. Complete blood count (CBC) revealed HGB 47 g/L [reference range 115-150 g/L]). MRI of the brain and whole spine showed abnormal signals of the cervical thoracic cord below the level of the spine centrum No. 6 and small circular hyper-intensity in the pituitary (Fig. 1). Bone marrow smears and peripheral blood smear implied megaloblastic change. Results of abdominal enhancement CT scanning, electronic gastroscopy, autoimmune antibodies, thyroid function and stool routine results were normal.

The primary diagnosis of this patient was SCD. We excluded the possibility of multiple sclerosis, compressive myelopathy, autoimmune and gastrointestinal diseases. The secondary diagnoses included organic mental disorder, megaloblastic anemia and Vitamin B12 deficiency.

The patient received intramuscular injection of Vitamin B12 500 mg per day. Folic acid was supplemented 10 mg orally 3 times per day. Vitamin B12-rich diet was recommended. We administered blood transfusion (3.5 units of suspended RBC) when found HBG of 47 g/L. Duloxetine 60 mg per day was used to improve depressive symptoms, with a combination of Olanzapine 10 mg per day which was administered for her psychiatric symptoms. Three days after caring in psychiatric quarantine ward, the patient increased inappetence. And her fear and suspicion worsened with persisting physical inability and maladaptation. Her vital signs remained stable. Her HGB increased to 84 g/L. A week later, her abnormal sensations, weakness, and numbness began to resolve, with fear and suspicion improving simultaneously. She achieved complete remission of her neurologic and psychiatric symptoms after 14 days of treatment. Eighteen days after admission, she was discharged with suggestions of supplementation of Vitamin B12, folic acid and other nutrients.

## Discussion and conclusion

SCD is a reversible neurodegenerative disease manifested as peripheral neuropathy damage, paresthesia, weakness, ataxia, deep sensitivity disturbances, and very rarely, optic atrophy and psychiatric symptoms [3]. However, studies focusing on the psychiatric symptoms among SCD patients are seldom reported. So far, we haven’t found any report about other possible causes of psychiatric symptoms except the direct physiological effects of SCD. Thus, we presented a case of a patient with SCD with psychiatric symptoms centering on physical discomfort to illustrate our opinions. The possible causes of her psychiatric symptoms were as follows.

Firstly, based on principle of monism diagnosis, both SCD and psychiatric symptoms are likely to be directly caused by Vitamin B12 deficiency instead of being irrelevant comorbidity. Her vegetarian diet for 8 years implied long-term influence from Vitamin B12 deficiency. Hallucinations, irritability, catatonia, delirium and apathy were commonly reported psychiatric symptoms caused by Vitamin B12 deficiency [2]. Vitamin B12 is a crucial coenzyme involving in the synthesis of myelin and neurotransmitters, the lack of which could interfere the metabolism of methionine and homocysteine, leading to cognitive impairment and psychiatric symptoms [4]. In addition, methylmalonyl-CoA cannot be converted into succinyl-CoA without Vitamin B12, because of which methylmalonic acid (MMA) accumulates and leads to demyelination of neurons [5]. These processes may lead to the appearance of psychiatric symptoms [6]. However, if so, the manifestations of psychological symptoms should be generalized, not characterized by depression and mood-congruent psychosis, nor should the psychiatric symptoms be centered on the lower extremities, which strongly overlapped with her physical suffering.

Another possibility to consider is the effects from anemia. The patient's HGB was already at a low level when the psychiatric symptoms appeared. The anemia may cause reduced oxygen-carrying capacity and insufficient energy supply to interfere brain metabolism [7, 8]. Anemia has been reported associated with psychiatric symptoms from mechanisms of genetic and autoimmune factors [9]. Nevertheless, the vital signs of this patient remained stable during the whole course and no signs of brain hypoxia such as drowsiness were noticed except irritability, which is still mood-congruent and non-specific. Also, both physical and psychological symptoms did not improve in 3 days after blood transfusion. Thus, this explanation is not plausible.

Thirdly, the persecutory delusions and delusions of being controlled were more likely to be secondary to abnormal body experiences like numbness and weakness. The psychiatric symptoms came after physical discomfort, which is time coherent. With undefined diagnosis and possible future of disability, it’s reasonable that the patient occurred psychological experience resembling that from trauma and stress. Moreover, with inability to take care of herself, decreased social contact, being always surrounded by her husband in daily life and medical staff after admission, plus our closed inpatient environment and still worsening physical condition, her fear was undoubtedly aggravated, leading to paranoid suspicion of everyone around her. Nonetheless, this supposition utilized a dualism perspective, where the physical mechanism is neglected.

Taking the 3 possible mechanisms in together, we guess it’s time to take a comprehensive thinking. These direct physiological effects of Vitamin B12 deficiency and anemia might have increased neurological and psychological vulnerability. Combined with severe physical manifestations on the lower extremities, we highlighted the possibility of psychiatric symptoms being caused by a combination of psychological and organic factors, where exacerbation of neurologic symptoms and rapid change of living conditions appeared to accelerate the development of psychiatric symptoms in this vulnerable individual. After physical recovery, the vulnerability increased, so the patient didn’t relapse at one-month follow-up while we already discontinued antipsychotics and antidepressants at discharge. The vulnerability perspective seems to be reasonable.

Our case also highlights the importance of evaluating Vitamin B12 levels in psychiatric patients, especially among high-risk populations such as vegetarians, the elderly, and patients with gastrointestinal disorders [10, 11]. The incidence of Vitamin B12 deficiency in psychiatric inpatients was found to be 6% in a general hospital [12]. Psychiatric disorders induced by Vitamin B12 must be rectified within 12 months to avoid irreversible damage [13].

To the best of our knowledge, we are the first to raise an argument that psychiatric symptoms in SCD patients could not only be generated from direct physiological effects of vitaminB12 deficiency but also associated with physical discomfort and environmental factors. We emphasized the importance of interdisciplinary consultation to get early detection and timely intervention of specialty diseases, which could effectively prevent treatment delay of primary disease and even the emergence of psychiatric symptoms. We reviewed the process of the patient's visit and found that although the medical workup did provide clues for diagnosis, the patient did not come for a scheduled follow-up visit and therefore did not receive systematic treatment until hospitalization. Confronting psychiatric symptoms combined with SCD, the use of antipsychotics and antidepressants is an additional key to improving the prognosis. Thus, a further systematical study is needed to confirm the effectiveness of different antipsychotics and antidepressants for treatments of SCD patients with psychiatric symptoms.

In conclusion, the psychiatric symptoms of SCD patient are attributable to a combination of Vitamin B12 deficiency, anemia, worsening physical condition, and personal and environmental disruption. Testing Vitamin B12 levels in patients with psychiatric symptoms, especially for whom with anemia, should not be neglected. The efficacy of antipsychotics and antidepressants in patients with SCD warrants further investigation.

## Data Availability

The data used during the current study are available from the corresponding author on reasonable request.

## Abbreviations

SCD: Subacute Combined Degeneration of the Spinal Cord
CT: Computed Tomography
MRI: Magnetic Resonance Imaging
CBC: Complete Blood Count
RBC: Red Blood Cell
HGB: Hemoglobin
MCV: Mean Corpuscular Volume
MMA: Methylmalonic Acid
